# Recombinant AAV-mediated *HSVtk *gene transfer with direct intratumoral injections and Tet-On regulation for implanted human breast cancer

**DOI:** 10.1186/1471-2407-6-66

**Published:** 2006-03-16

**Authors:** LI Zi-Bo, ZENG Zhao-Jun, CHEN Qian, LUO Sai-Qun, HU Wei-Xin

**Affiliations:** 1 Molecular Biology Research Center, Xiangya Medical College, Central South University, 110 Xiangya Road, Changsha, Hunan 410078, P. R. China

## Abstract

**Background:**

HSVtk/ganciclovir (GCV) gene therapy has been extensively studied in tumors and relies largely on the gene expression of *HSVtk*. Most studies, however, have failed to demonstrate any significant benefit of a controlled gene expression strategy in cancer treatment. The Tet-On system is commonly used to regulate gene expression following Dox induction. We have evaluated the antitumor effect of HSVtk/ganciclovir gene therapy under Tet-On regulation by means of adeno-associated virus-2 (AAV-2)-mediated *HSVtk *gene transfer with direct intratumoral injections in mice bearing breast cancer tumors.

**Methods:**

Recombinant adeno-associated virus-2 (rAAV) was constructed and transduced into MCF-7 cell line. GCV treatment to the rAAV infected MCF-7 cells was performed by MTT assay under the doxycycline (Dox) induction or without Dox induction at a vp (viral particle) number of ≥10^4 ^/cell. The virus was administered intratumorally to nude mice that had also received GCV intraperitoneally. The antitumor effects were evaluated by measuring tumor regression and histological analysis.

**Results:**

We have demonstrated that GCV treatment to the infected MCF-7 cells under the Dox induction was of more inhibited effects than those without Dox induction at ≥10^4 ^vp/cell. In *ex vivo *experiments, tumor growth of BALB/C nude mice breast cancer was retarded after rAAV-2/HSVtk/Tet-On was injected into the tumors under the Dox induction. Infiltrating cells were also observed in tumors after Dox induction followed by GCV treatment and cells were profoundly damaged. The expression of *HSVtk *gene in MCF-7 cells and BALB/C nude mice tumors was up-regulated by Tet-On under Dox induction with reverse transcription-PCR (RT-PCR) analysis.

**Conclusion:**

The antitumor effect of rAAV-mediated HSVtk/GCV gene therapy under the Dox induction with direct intratumoral injections may be a useful treatment for breast cancer and other solid tumors.

## Background

For enhancing gene therapeutic effects of breast cancer, there are several approaches including the inhibition of oncogene functioning [1], the rehabilitation of the tumor suppressor genes [2], enhancement of the immune response of tumor cells or immunological cells [3,4], increase in the immunological tolerance at chemotherapy of normal cells [5], suppression of tumor angiogenesis [6], and the transformation of some apoptotic genes into tumor cells [7], as well as suicide gene therapy [8]. The last approach, which can offer the prospect of selectively introducing genes into cancer cells, rendering them susceptible to specific antitumor drugs, is especially appealing for this bystander effect [9]. This is significant since the high therapeutic index that can be achieved with a high concentration of toxic drug will occur only at the tumor site. The more widely used suicide gene system is the herpes simplex virus thymidine kinase/ganciclovir (HSVtK/GCV). GCV, a kind of antiviral drug, can be phosphorylated by HSVtK protein finally into triphosphates, which are potent inhibitors of DNA polymerase, leading to the disruption of cellular DNA synthesis and ultimately cell death. These properties make the system attractive in cancer therapy [9,10].

The progress was made in increasing the product of therapeutic genes combined with suicide gene therapy using either tumor cell-specific or tissue-specific promoters. Both are capable of directing expression of a heterologous gene in tumor cells [11-13]. However, only a few workers have reported precise regulation of the *HSVtk *gene. Recent studies have also shown that a specific dose of the therapeutic gene product is required for successful treatment of certain disease, but excessive production of the gene product may be toxic in some cases [14,15]. For better treatment, it may be necessary to control the product of therapeutic genes within a window of appropriate time and dose. The Tet-On regulatable system can tightly regulate gene expression in response to induction and be switched off when necessary, which is of value in gene therapy [16]. The system is based on the interaction between the tetracycline-responsive element (TRE), consisting of seven copies of the prokaryotic tetracycline operator site (tetO) fused to a cytomegalovirus minimal promoter region, and the reverse tetracycline-transactivator (rtTA), consisting of the prokaryotic tetracycline repressor protein (TetR) fused to the activator domain of the herpes simplex virus VP16 protein. In the presence of tetracycline (tet) or its derivative Dox, rtTA binds TRE and activates gene expression. Compared with several drug-dependent regulatable systems, the Tet-On system shows several superior characteristics for applications in patients, including high induction, rapid reaction and high specificity [17,18]. This system had been studied in the context of numerous viral and non-viral vectors to regulate expression of various genes, and diseases needed intermittent treatment [19].

For suicide gene therapy, the transfer of therapeutic genes into the tumor cells and the maintenance of their expression in tumors require efficient vector systems. The adeno-associated viral (AAV) is a promising gene transfer vector for human gene therapy because of its unique combination of attractive properties. These include: (1) the efficiency of in vivo gene transfer to a number of different cell types, including skeletal muscle cells, hepatocytes, neurons, and photoreceptors; (2) the ability to transduce dividing and growth arrested cells; (3) the availability of procedures for high-titre, helper virus-free preparation; and (4) a lack of significant toxicity or immune response. Furthermore, AAV-mediated gene delivery produces a long-term gene expression following prenatal administration and after administration in adult tissues [20,21].

In our previous study, the pseudoviruses, pRev*TRE/HSVtk *and pRev*Tet-On*, in which the expression of suicide tk gene is tightly controlled by Dox, were used to infect MCF-7 cells or were directly delivered into the model of BALB/c nude mice bearing human breast cancer. The findings were that *HSVtk *gene expression was controlled by the Dox-induced Tet-On system, and it is feasible for treating human breast cancer [22,23]. However, TRE and rtTA were respectively cloned into two vectors, which were transferred into host cells at their appropriate ratios. There is a latent danger in insertion mutation and activation of oncogenes in human being using retroviral vector system [24]. We explored the treatment of human breast cancer by AAV-2 expressing *HSVtk *gene under the regulation of *Tet-On*, the rAAV (rAAV-2/*TRE/HSVtk*/*Tet-On*) was constructed and transduced into MCF-7 cell line or BALB/c nude mice. In order to test the antitumor effect with this system, nude mice bearing breast cancer were treated with GCV followed by rAAV intratumoral injection and consecutive induction by Dox. This antitumor treatment may also offer a new approach and ideal model in human breast cancer or other solid tumor gene therapy.

## Methods

### Cell line, cell culture and vectors

Packaging cell line 293 (ATCC # CRL-11268™), human breast cancer cell line MCF-7 (ATCC # HTB-22™) and mouse fibroblast cell line NIH 3T3 (ATCC # CRL-1658™) originated from the American Type Culture Collection (ATCC, Manassas, VA). Cells were cultured in Dulbecco's modified Eagle's medium (DMEM) supplemented with 10 % of fetal bovine serum (FBS) plus 100 units of penicillin per ml, and 0.1 mg of streptomycin per ml. The cells were grown at 37°C in a humidified incubator with an atmosphere of 5 % CO_2_. The AAV-2 Helper-Free system, including the plasmids of pAAV-MCS, pAAV-RC, pAAV-Helper, came from Stratagene (La Jolla, California, USA). The RevTet-On system, including the plasmids of pRev*TRE *and pRev*Tet-On*, was supplied by Clontech (Palo, Alto, CA).

### The construction of pAAV/TRE/HSVtk/*Tet-On*

pRev*TRE/HSVtk *was constructed as previously described [22]. In brief, *Tet-On *gene fragment was amplified by PCR technique with primers Tet1/Tet2 (Tet1 5 ' -CTC T**AT CGA T**GG ATC CGG CCA TTA GCC AT-3'; Tet2 5 ' -CTC T**GT TAA C**TA CCC ACC GTA CTC GTC-3'; Cla I and Hpa I restriction sites are shown in boldface) from the plasmid pRev*Tet-On*. The PCR product was recovered from agarose gel and digested with *Cla *I and *Hpa *I. The recovered DNA fragment was inserted into pRevTRE/HSVtk to produce the recombinant plasmid pRev*TRE*/*HSVtk*/*Tet-On*. The fusion DNA fragment of *HSVtk *and *Tet-On *was cut with *Xho *I and *Cla *I and inserted into pAAV-MCS. The recombinant plasmid pAAV/*TRE/HSVtk/Tet-On *was constructed followed by partial digestion of *Not *I and *Cla *I, filling the 3' end and self-ligation.

### Preparation, purification and titre detection of rAAV

Packaging cells 293 were transfected by the method of modified calcium phosphate co-precipitation with pAAV/*TRE/HSVtk*/*Tet-On*, pAAV-RC and pAAV-Helper when the cell monolayers were 50–60% confluent. The cells were collected after 72 days of transfection, and resuspended in cell lysis buffer (10 mM Tris-HCl, pH 8.5, 150 mM NaCl). The cells were frozen and thawed 3 times in liquid nitrogen and 37°C respectively. The lysate was centrifuged at 4,000 g for 10 min and the supernatant was collected. Solid NaCl and PEG-8000 were added in supernatant to reach concentrations of 1 M and 10% (W/V) respectively, and the mixture agitated to dissolve these components whilst being incubated on ice for 1 h. After 9,000 g centrifugation for 15 min, the pellet was mixed with 1.38 g/ml of CsCl_2 _and centrifuged at 500,000 g for 24 h at 4°C to isolate the AAV-2/*TRE/HSVtk*/*Tet-On *viral particles. Titre detection was performed by dot blot hybridization for purified rAAV. The physical titre of AAV-2/*TRE/HSVtk/Tet-On *virus was 2.38 × 10^11 ^viral particle/ml.

### rAAV infections and MTT assay

MCF-7 cells were cultured at 2 × 10^3 ^cells per well in 96-well plates. The experimental cultures were divided into 3 groups, to which 2 × 10^7^, 2 × 10^8 ^and 2 × 10^9 ^vp of rAAV-2/*TRE/HSVtk*/*Tet-On *were added per well, at 37°C for 8 h. The ratio of viral particles and cells was as 10^4 ^vp/cell, 10^5 ^vp/cell, and 10^6 ^vp/cell. Twenty-four hours later, 1.0 μg/ml Dox and 3.0μg/ml GCV (Sigma, St. Louis, MO) were added to each well of group 1, 3.0 μg/ml GCV was added to each well of group 2, and 1.0 μg/ml Dox was added to each well of group 3. Cell viability was determined by the 3-(4, 5-dimethylthiazol-2-yl)-2, 5-diphe nyltetrazolium bromide (MTT, Sigma) assay after another 72 h incubation. The cells were incubated with 0.5% MTT for 4 h. The medium was then removed and 100 μl of 0.04 M dimethyl sulfoxide solution added, followed by incubation at 37°C for a further 4 h. The absorbance of the reaction product in solution at 570 nm was measured. The data were expressed as mean ± S.D and used to draw growth curves. To calculate the percentage of living cells, we divided the cell number in the experimental wells by the cell number in the control wells (no Dox or no GCV). The growth curves were protracted by a manual count method. After 48 h culture, viable cells were counted by the trypan blue dye exclusion method. All experiments were performed 4 times independently.

### Detection of *HSVtk *gene expression

Expression of *HSVtk *gene in the infected MCF-7 cells and tumor tissues of BALB/c nude mice was analyzed by RT-PCR. 0.1 g of fresh tumor tissue with 1 ml of pre-cooled TRIzol reagent (Invitrogen, La Jolla, CA) was homogenized and total RNA was extracted according to instruction. RT-PCR was performed with nested primers *TK3/TK4 *(*TK3*: 5'-TGG AGC AGA AAA TGC CCA CG-3' *TK4*: 5'-TGC TGC CCA TAA GGT ATC GC-3') (a DNA fragment of 420 bp). For internal control, *β-actin *(sense: 5'-TGA CGG TCA GGT CAT CAC TAT CGG CAA TGA-3' and antisense: 5'-TTG ATC TTC ATG GTG (AGC) TAG GAG CGA GGG CA-3') were co-amplified with *HSVtk *(a DNA fragment of 260 bp). Before reverse transcription (RT), residual genomic DNA was removed by treatment with RQ DNase (Promega, Madison, WI). Total RNA (1.5 μg) was used to synthesize cDNA in 20 μl of reaction mixture by standard protocol with oligo(dT)_15 _as primer, and AMV reverse transcriptase (Promega). PCR reactions were carried out with 5 μl of RT products in 50 μl of reaction system using *Taq *DNA polymerase. After an initial denaturation 94°C for 5 min, 30 cycles of amplification were performed under following condition: 94°C for 30 seconds, 56°C for 45 seconds, and 72°C for 40 seconds. PCR products were run on 1.5 % agarose gel (containing 0.5 mg of ethidium bromide per liter).

### Establishment of human breast cancer models in BALB/c nude mice and GCV treatment

Female BALB/c nude mice, 4–5 week-old and weighing 20.0 ± 2.0 grams, came from the Laboratory Animal Center of the Academy of Medical Sciences of China (Shanghai, China). The mice were housed in vinyl cages equipped with air filter lids, which were kept in laminar airflow hoods and maintained under pathogen-limiting conditions. All mice were cared for according to institutional guidelines, and all food, water, caging, and bedding were sterilized before use. All procedures were approved by the National Animal Care and Use Committee in China and the Laboratory Animal Center of the Academy of Medical Sciences of *Xiangya Medical College *granted approval for the experimental research on animals. BALB/c nude mice were given a single priming dose of cyclophosphamide (100 mg/kg body weight, subcutaneously [s.c.]) 3 days prior to inoculation of cells to deplete the natural killer cells of mice. MCF-7 cells were trypsinized, washed with DMEM 3 times to remove enzyme, and harvested by centrifugation. Cell pellets were resuspended in serum-free DMEM. After a short period of ether inhalation anesthesia by nude mice, 2 × 10^7 ^cells in 0.2 ml of suspension were injected s.c. into the left flank regions to produce tumors. Tumor size was measured every 3 days with vernier caliper. Thirty-two mice were randomly divided into 4 groups: GCV + Dox (8 mice), GCV + rAAV (8 mice), GCV + Dox + rAAV (8 mice), DMEM only (8 mice). When tumors had reached 7–8 mm in diameter, intratumoral injection of AAV-2/*TRE/HSVtk*/*Tet-On *(100 μl, 2 × 10^11 ^vp/ml) or DMEM (mock group) was performed 4 times (twice a week). Tumors in mice were induced by Dox (20 mg/kg) for 1 week prior to intraperitoneal administration of GCV (100 mg/kg) twice daily for 14 days. The volumes of tumor were calculated using the following formula: volume V = L × W^2 ^π/2, where L is the length and W is the width of the tumor (in millimeters).

### Histological analysis

Subcutaneous tumors were excised from BALB/c nude mice with different treatments. Tumors were rinsed twice in normal saline (NS) then fixed in 4% polyparaformaldehyde for 12 h, embedded in paraffin and sections were stained with hematoxylin and eosin for light microscopic observation.

### Statistical analysis

Results are expressed as means ± standard deviation (S.D). The statistical difference between the groups was determined by one-way analysis of variance test. The software for statistics is SPSS (10.0 version). Differences among groups were considered statistically different at *P <*0.05.

## Results

### Killing effect of *HSVtk *on MCF-7 cells under the induction of Dox

**Figure 1:**
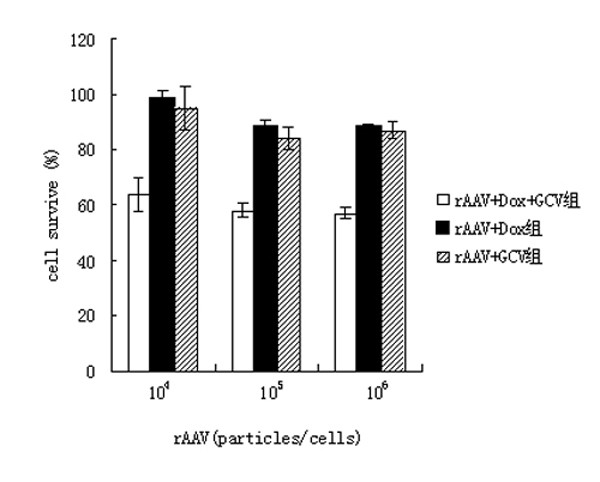
**Effects of rAAV/*TRE/HSVtk/Tet-On *on MCF-7 cells in the presence of GCV followed by the induction of Dox**. MCF-7 cells were transduced with various doses of rAAV/*TRE/HSVtk*/*Tet-On*. Twenty-four hours after transduction, the rAAV+Dox+GCV group (blank bar) cells were exposed to 3.0 μg/ml GCV and 1.0 μg/ml Dox while the rAAV+Dox group (black bar) cells with 1.0 μg/ml Dox and the rAAV+GCV group (hatched bar) cells with 3.0 μg/ml GCV. After 3 days, the survival rate was calculated as described in Materials and Methods. Data showed that the survival rate of the rAAV+Dox+GCV group cells was different from that of the other two groups cells. Values are expressed as means ± SD.

MCF-7 cells in 96-well plate were infected with 10^4 ^vp/cell, 10^5 ^vp/cell and 10^6 ^vp/cell of AAV-2/*TRE/HSVtk*/*Tet-On *respectively. After induction of 1.0 μg/ml Dox, the killing rate of rAAV+Dox+GCV with 10^4 ^vp/cell to MCF-7 reached 36%, as detected by using the MTT assay in the presence of GCV (3.0 μg/ml). Increasing the viral titre to 10^6 ^vp/cell raised the cell death rate to 47% (Fig. 1). Almost no difference was found between MCF-7 cells survival as rAAV infection reached a plateau when rAAV titres ranged from 10^4 ^to 10^6^vp/cell. Although CsCl_2 _density gradient ultracentrifugation procedure allows for the enrichment of pure and packaged AAV-2 vector particles, it often produces vector preparations of variable quality, and may lead to a loss of particle infectivity [21]. We also indirect tested the transduction efficiency with pAAV-LacZ according to the *Stratagene *protocol. In our experiment, the efficiency of AAV infection to MCF-7 cells is 15–20%. In all kinds of rAAV titres above, the cell death rate of rAAV+Dox+GCV group was obviously higher than rAAV+GCV group and rAAV+Dox group (*P *< 0.05).

### GCV treatment for implanted human breast cancers in vivo

**Figure 2:**
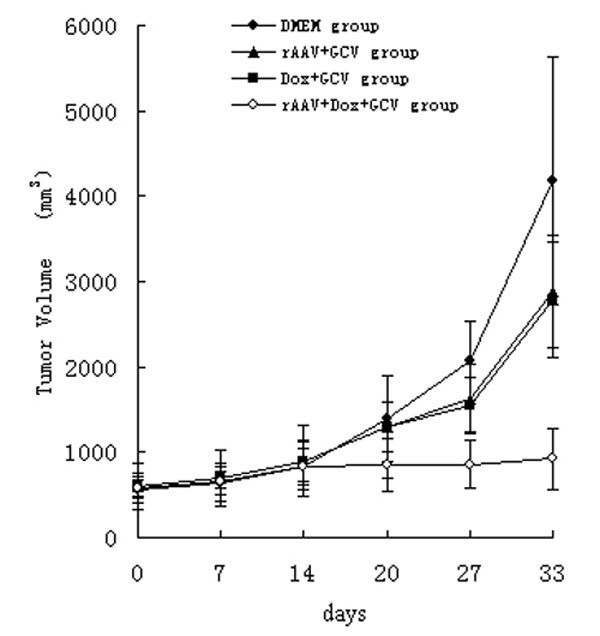
**Tumor volume analysis after treatment in BALB/c nude mice**. Implanted tumors were generated by injecting MCF-7 cells in the flank in BALB/c nude mice. When subcutaneous tumors reached approximately 7–8 mm in diameter, rAAV/*TRE/HSVtk*/*Tet-On *virus (2 × 10^11 ^particles/ml) or mock-infection (DMEM) were performed direct intratumor injection to human breast cancer model on day 0, followed by a second injection on day 7. GCV treatment (100 mg/kg) was started on day 0 and continued for another 14 days after induced by Dox for one week. Tumor size was measured every 3 days. Tumor volume values (mm^3^) are expressed as means ± SD. The tumors of the rAAV/tk/On+Dox+GCV group demonstrated significant tumor growth suppression after GCV treatment compared with mock (*P *< 0.05 on day 30 after infection).

During the first 14 days of GCV treatment, tumor volume in all groups increased at almost the same rate. However, in the GCV + Dox + virus treated group, tumor growth rate was retarded after 30 days compared with the control groups (Fig. 2). In the controls, tumors grew continuously up to 2 cm in diameter by the end of the 40th day when the mice were killed. For tumors injected with rAAV, GCV treatment after the induction of Dox resulted in suppression of all tumors by day 30 (Fig. 2). The suppression rate of xenograft tumor growth reached up to 84%, and showed a strong inhibitory effect on tumor growth in vivo. These data suggested that *HSVtk *gene was expressed in a regulated manner in implanted human breast cancer infected intratumorally with rAAV.

### Histological analysis of human breast cancer in BALB/c nude mice

**Figure 3:**
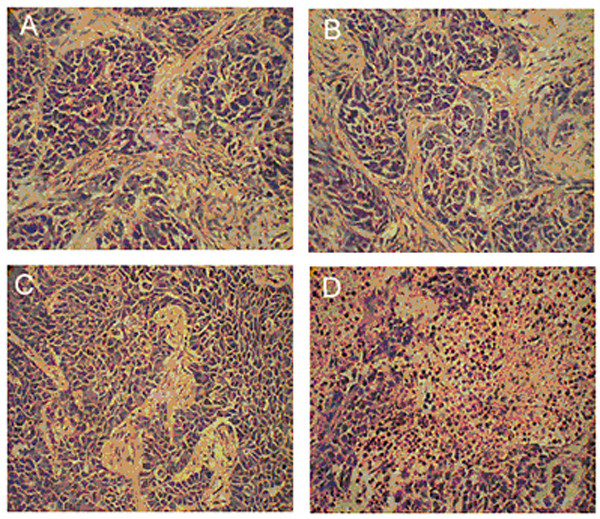
**Tumor slices stained with hematoxylin and eosin**. Subcutaneous tumors were excised from BALB/c nude mice with different treatments under sodium pentobarbital (50 mg/kg, i.p.) anesthesia. Tumors were rinsed twice in normal saline (NS) then fixed in 4% polyparaformaldehyde for 12 hours, embedded in paraffin, sliced and sections stained with hematoxylin and eosin for light microscopic observation (× 400 magnification). A: DMEM group; B: GCV+Dox group; C: GCV+Virus group; D: Dox+GCV+Virus group.

BALB/c nude mice bearing human breast cancer with different treatments were given sodium pentobarbital (50 mg/kg, i.p.) anesthesia to excise the subcutaneous tumors. In the hematoxylin and eosin stained sections, few accompanying infiltrating cells were seen in control groups. On the other hand, remarkable levels of infiltration of lymphocytes were observed throughout the tumors that had been injected with Dox followed by GCV treatment, and breast cancer cells were severely damaged (Fig. 3).

### HSVtk gene expression in tumor tissue and rAAV-infected MCF-7 cells

**Figure 4:**
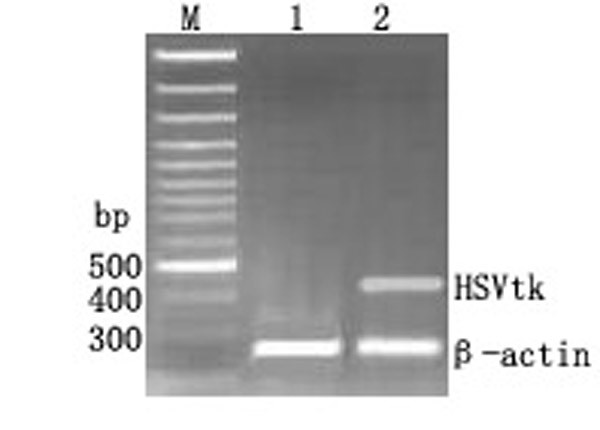
**MCF-7 cells were infected with 10^5 ^vp/cell of rAAV/*TRE/HSVtk/Tet-On***. Twenty-four h after transduction, one group was exposed to 10 μg/ml of Dox and the other group without Dox administration. After 3 days, RNAs were extracted from the 2 groups and *HSVtk *gene expression was detected by RT-PCR with nested primers TK3/TK4 (a DNA fragment of 420 bp). For internal control, β-actin was co-amplified with *HSVtk*. The amplification product of *HSVtk *gene was observed in Dox inducement group but not in without inducement group. M: 100 bp DNA ladder; 1:group without induction; 2:group inducted with 1 g/L Dox

**Figure 5:**
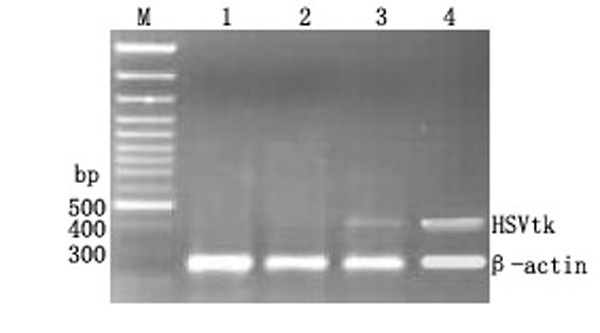
***HSVtk *gene expression level in implanted tumors of BALB/c nude mice and detection by RT-PCR**. Total RNA was isolated from implanted tumors of nude mice.*HSVtk *gene expression level was detected by RT-PCR with the nested primers TK3/TK4 (a DNA fragment of 420 bp). For internal control, β-actin was amplified together with *HSVtk*. The amplification product of *HSVtk *gene was observed in the Dox+GCV+virus group and the weak signal was detected in GCV+virus group. M: 100 bp Marker; 1: DMEM group; 2: GCV+Dox group; 3: GCV+virus group; 4: Dox+GCV+virus group.

In order to evaluate the efficiency of gene transfer in vivo, we used RT-PCR to detect the expression of *HSVtk *gene. A 420 bp fragment of *HSVtk *gene was observed, which was consistent with predicted results (Fig. 4). We detected high level of expression of *HSVtk *in tumor tissues of GCV + Dox+ rAAV treated group. The faintest band could be detected in the rAAV+GCV group and no band in other two groups (the DMEM group and the Dox+GCV group) could be seen (Fig. 5). After induction of Dox, the *HSVtk *expression level was enhanced by approximately fourfold in tumors subjected to treatment with GCV and viruses. This result was agreement with the growth of breast cancer in mice. As expected, without *HSVtk *expression, the growth of parental MCF-7 cells was not affected by GCV and tumors continued to grow until the mice were killed because of large tumor loads. A similar result was obtained in rAAV-infected MCF-7 cells.

## Discussion

We constructed rAAV vector, which contained *Tet-On *regulatory sequences and the *HSVtk *gene, in an effort to develop a novel treatment for breast cancer and other solid tumors. Our results demonstrate that this vector is capable of directing expression of the *HSVtk *gene in breast cancer and results in suppression of tumor growth rate, when delivered by direct intratumoral injection and induced by appropriate Dox with conjunction of GCV. Our results also suggest that direct intratumoral injection is not only a simple and practical gene delivery method in local cancer gene therapy, but that it also avoids systemic toxicity [25]. All the cancerous nude mice survived longer after the treatment, and toxicity of the treatment in mice was acceptable.

In cancer gene therapy, viral vectors are the most efficient means of delivering a corrective gene into human cells, such as retroviral vectors and adenoviral vectors. However, the applications of retroviral vectors for gene therapy face a number of challenges. Retroviral vectors has some disadvantage of infection only dividing cells, randomly integration of therapeutic gene, risk of insertional mutagenesis and activation of oncogenes, which correlates with tumor in human being [24]. The adenovirus vector is potentially of a strong immunogenic character and has a low transduction efficiency in solid tumor [26,27]. AAV can integrate into the specific site of human chromosome 19 and is not pathogenic for human being, especially without the risk of tumorigenesis. MCF-7 cells were highly infected and expressed by recombinant AAV-2 vector in study of Veldwijk [28]. Due to the small fragment of the *Tet-On *regulatory units, AAV-2 vector was allowed to introduce the *HSVtk *gene although it possesses about 5 Kb capability. The tetracycline-responsive system is an ideal system for tightly regulation gene expression mediated by rAAV [29], because it is the tet responsive cassette inducible unit whose activity is modified in response to Dox. In rAAV vector we constructed, TRE and rtTA units of *Tet *gene regulation system were inserted inversely each other, to delete the downstream activity of the *HSVtk *gene by rtTA gene promoter through antisense suppression and subtraction.

Tumor growth in nude mice given our treatment was inhibited but not eradicated. It is quite difficult to eradicate the tumors treated by one kind of a single gene since tumorigenesis and development is a complicated progress involving multigenes [30]. In order to enhance the efficiacy of the treatment, some approaches were adopted, such as combination of two suicide genes or combination with other therapies [30,31]. Suicide gene therapy in combination with other methods, such as adjuvant radiotherapy or chemotherapy, may have some promising use in the clinic because it does not produce any elevation of the toxic side-effects in breast cancer treatment as it reaches drug concentration high enough to cure local tumors [32,33]. For some long period and progressive diseases, such as diabetes or cancer, the control of therapeutic genes expression is of great importance in this gene therapy. However, intrinsic to lengthy gene therapy treatment is the need to alter expression in response to disease progression, growth and development of younger patients, or untoward side-effects. Regulating gene expression by exogenously administered drugs provides control over the level of functional gene product. Regulation permits much greater control over timing of gene expression and can provide a baseline level of behavior or cellular function in the presence of Dox. To have control over the time and level of gene expression is essential for both the in vitro and in vivo studies of gene function, especially for the safety and efficacy of gene therapy.

The *HSVtK *gene expression in tumor cells of nude mice was detected by RT-PCR and there was a faint *HSVtK *gene expression without Dox induction, as shown in our data. It may be due to leakage of the Tet-On regulatory system [34], but this does not influence the efficiency of the treatment as it has only lower suicide gene expression compared with the experimental group. Sometimes the therapeutic gene is still expressed when Dox does not exist because there is a residual affinity between TRE and rtTA units of the system. So many measures are taken using a certain modified rtTA system which has shown promising results in low basal expression in the absence of inducer, high induced expression, high induction ratio, indifference to endogenous cellular processes, low immunogenicity of system components and safety of inducers [35-38].

We constructed rAAV vectors containing Tet-On system and *HSVtk *gene to explore the effect of breast cancer treatment. Our results show that the expression of *HSVtk *gene was controlled by Tet-On system under the induction of Dox, transduced by rAAV. The antitumor treatment occurred with an acceptable toxicity level, and the treated mice achieved long-term survival. The effectiveness of the *HSVtk*/*Tet-On*/GCV system would provide an effective regulatory treatment strategy for breast cancer including other solid tumors.

## Conclusion

In conclusion, our data demonstrated that tumor growth of BALB/C nude mice breast cancer was inhibited with direct intratumoral injections of rAAV-2/HSVtk/Tet-On under the Dox induction. The expression of *HSVtk *gene in MCF-7 cells and BALB/C nude mice tumors was up-regulated by Tet-On under Dox induction. Our studies suggest that rAAV-mediated HSVtk/GCV gene therapy under the Dox induction with direct intratumoral injections may be a useful method in treatment for breast cancer and other solid tumors.

## Abbreviations

AAV (adeno-associated virus); *HSVtk *(herpes simplex virus thymidine kinase); GCV (ganciclovir); Dox (doxycycline); v.p (viral particle); rtTA (reverse tetracycline transactivator); TRE (tetracycline-responsive element); tet (tetracycline); DMEM (Dulbecco's modified Eagle's medium); FBS (fetal bovine serum).

## Competing interests

The author(s) declare that they have no competing interests.

## Authors' contributions

LI Zi-Bo is responsible for the generation of rAAV particles expressing HSVtK and Tet-On, rAAV infections and MTT assay. ZENG Zhao-Jun carried out animal experiments and contributed to the evaluation of treatment effects and is co-first author. CHEN Qian and LUO Sai-Qun contributed to the construction of rAAV vector. HU Wei-Xin, as the corresponding author, designed the protocol and made the draft of the manuscript. All authors read and approved the final manuscript.

## Pre-publication history

The pre-publication history for this paper can be accessed here:



## References

[B1] Palmer K, Sharan N, Emtage P, Gauldie J, Muller WJ, Wan Y (2002). Intratumoral administration of an adenovirus expressing a kinase dead form of ErbB-2 inhibits tumor growth. Gene Therapy.

[B2] Sevignani C, Calin GA, Cesari R, Sarti M, Ishii H, Yendamuri S, Vecchione A, Trapasso F, Croce CM (2003). Restoration of fragile histidine triad (FHIT) expression induces apoptosis and suppresses tumorigenicity in breast cancer cell lines. Cancer Research.

[B3] Janat-Amsbury MM, Yockman JW, Lee M, Kern S, Furgeson DY, Bikram M, Kim SW (2004). Combination of Local, Nonviral IL12 Gene Therapy and Systemic Paclitaxel Treatment in a Metastatic Breast Cancer Model. Molecular Therapy.

[B4] Nakamori M, Fu X, Rousseau R, Chen SY, Zhang X (2004). Destruction of nonimmunogenic mammary tumor cells by a fusogenic oncolytic herpes simplex virus induces potent antitumor immunity. Molecular Therapy.

[B5] Cowan KH, Moscow JA, Huang H, Zujewski JA, O'Shaughnessy J, Sorrentino B, Hines K, Carter C, Schneider E, Cusack G, Noone M, Dunbar C, Steinberg S, Wilson W, Goldspiel B, Read EJ, Leitman SF, McDonagh K, Chow C, Abati A, Chiang Y, Chang YN, Gottesman MM, Pastan I, Nienhuis A (1999). Paclitaxel chemotherapy after autologous stem-cell transplantation and engraftment of hematopoietic cells transduced with a retrovirus containing the multidrug resistance complementary DNA(MDR1) in metastatic breast cancer patients. Clinical Cancer Research.

[B6] Li H, Lindenmeyer F, Grenet C, Opolon P, Menashi S, Soria C, Yeh P, Perricaudet M, Lu H (2001). AdTIMP22 inhibits tumor growth, angiogenesis, and metastasis, and prolongs survival in mice. Human Gene Therapy.

[B7] Lin T, Zhang L, Davis J, Gu J, Nishizaki M, Ji L, Roth JA, Xiong M, Fang B (2003). Combination of TRAIL gene therapy and chemotherapy enhances antitumor and antimetastasis effects in chemosensitive and chemoresistant breast cancers. Molecular Therapy.

[B8] Stribbling SM, Friedlos F, Martin J, Davies L, Spooner RA, Marais R, Springer CJ (2000). Regressions of established breast carcinoma xenografts by carboxypeptidase G2 suicide gene therapy and the prodrug CMDA are due to a bystander effect. Human Gene Therapy.

[B9] Asklund T, Appelskog IB, Ammerpohl O, Langmoen IA, Dilber MS, Aints A, Ekstrom TJ, Almqvist PM (2003). Gap junction-mediated bystander effect in primary cultures of human malignant gliomas with recombinant expression of the *HSVtk *gene. Experimental Cell Research.

[B10] Beltinger C, Fulda S, Kammertoens T, Uckert W, Debatin KM (2000). Mitochondrial amplification of death signals determines thymidine kinase/ganciclovir-triggered activation of apoptosis. Cancer Research.

[B11] Yu L, Yamamoto N, Kadomatsu K, Muramatsu T, Matsubara S, Sakiyama S, Tagawa M (2004). Midkine promoter can mediate transcriptional activation of a fused suicide gene in a broader range of human breast cancer compared with c-erbB-2 promoter. Oncology.

[B12] Qiao J, Doubrovin M, Sauter BV, Huang Y, Guo ZS, Balatoni J, Akhurst T, Blasberg RG, Tjuvajev JG, Chen SH, Woo SL (2002). Tumor-specific transcriptional targeting of suicide gene therapy. Gene Therapy.

[B13] Maeda T, O-Wang J, Matsubara H, Asano T, Ochiai T, Sakiyama S, Tagawa M (2001). A minimum c-erbB-2 promoter-mediated expression of herpes simplex virus thymidine kinase gene confers selective cytotoxicity of human breast cancer cells to ganciclovir. Cancer Gene Therapy.

[B14] A-Mohammadi S, Hawkins RE (1998). Efficient transgene regulation from a single tetracycline- controlled positive feedback regulatory system. Gene Therapy.

[B15] Hillman GG, Slos P, Wang Y, Wright JL, Layer A, De Meyer M, Yudelev M, Che M, Forman JD (2004). Tumor irradiation followed by intratumoral cytokine gene therapy for murine renal adenocarcinoma. Cancer Gene Therapy.

[B16] Gossen M, Freundlied S, Bender G, Muller G, Hillen W, Bujard H (1995). Trandcriptional activation by tetracyclines in mammalian cells. Science.

[B17] Aurisicchio L, Bujard H, Hillen W, Cortese R, Ciliberto G, La Monica N, Palombo F (2001). Regulated and prolonged expression of mIFN(α) in immunocompetent mice mediated by a helper-dpendent adenovirus vector. Gene Therapy.

[B18] Zabala M, Wang L, Hernandez-Alcoceba R, Hillen W, Qian C, Prieto J, Kramer MG (2004). Optimization of the Tet-on system to regulate interleukin 12 expression in the liver for the treatment of hepatic tumors. Cancer Research.

[B19] Chtarto A, Bender HU, Hanemann CO, Kemp T, Lehtonen E, Levivier M, Brotchi J, Velu T, Tenenbaum L (2003). Tetracycline-inducible transgene expression mediated by a single AAV vector. Gene Therapy.

[B20] Gafni Y, Pelled G, Zilberman Y, Turgeman G, Apparailly F, Yotvat H, Galun E, Gazit Z, Jorgensen C, Gazit D (2004). Gene therapy platform for bone regeneration using an exogenously regulated, AAV-2-based gene expression system. Molecular Therapy.

[B21] Grimm D, Kleinschmidt JA (1999). Progress in adeno-associated virus type 2 vector production: promises and prospects for clinical use. Human Gene Therapy.

[B22] Hu WX, Zeng ZJ, Luo SQ, Chen Q (2004). Suicide gene therapy of human breast cancer in SCID mice model by the regulation of Tet-On. Chinese Medical Journal.

[B23] Zeng ZJ, Li ZB, Luo SQ, Hu WX (2006). Retrovirus- mediated tk gene therapy of implanted human breast cancer in nude mice under the regulation of Tet-On. Cancer Gene Therapy.

[B24] McTaggart S, Al-Rubeai M (2002). Retroviral vectors for human gene delivery. Biotechnology Advances.

[B25] Wang Y, Hu JK, Krol A, Li YP, Li CY, Yuan F (2003). Systemic dissemination of viral vectors during intratumoral injection. Molecular Cancer Therapy.

[B26] Berlinghoff S, Veldwijk MR, Laufs S, Maser HP, Jauch A, Wenz F, Jens Zeller W, Fruehauf S (2004). Susceptibility of mesothelioma cell lines to adeno-associated virus 2 vector-based suicide gene therapy. Lung Cancer.

[B27] Enger PO, Thorsen F, Lonning PE, Bjerkvig R, Hoover F (2002). Adeno-associated viral vectors penetrate human solid tumor tissue in vivo more effectively than adenoviral vectors. Hum Gene Therapy.

[B28] Veldwijk MR, Fruehauf S, Schiedlmeier B, Kleinschmidt JA, Zeller WJ (2000). Differential expression of a recombinent adenoassociated virus 2 vector in human CD34^+ ^cells and breast cancer cells. Cancer Gene Therapy.

[B29] Fitzsimons HL, McKenzie JM, During MJ (2001). Insulators coupled to a minimal bi-directional tet cassette for tight regulation of rAAV-mediated gene transfer in the mammalian brain. Gene Therapy.

[B30] Lee KH, Piao H, Son BR, Heo DS, Kim NK, Kim ST (2004). Herpes simplex virus thymidine kinase and granulocyte marcrophage colony-stimulating factor combination gene therapy in a murine CT26 cell colon cancer model. Cancer Gene Therapy.

[B31] Goto T, Nishi T, Kobayashi O, Tamura T, Dev SB, Takeshima H, Kochi M, Kuratsu J, Sakata T, Ushio Y (2004). Combination electro-gene therapy using herpes virus thymidine kinase and interleukin-12 expression plasmids is highly efficient against murine carcinomas in vivo. Molecular Therapy.

[B32] Sobotkova E, Duskova M, Smahel M, Holan V, Janouskova O, Vonka V (2004). Chemotherapy and immunotherapy of tumours induced by gene-modified HPV16-transformed cells. Oncology Reports.

[B33] Nestler U, Wakimoto H, Siller-Lopez F, Aguilar LK, Chakravarti A, Muzikansky A, Stemmer-Rachamimov A, Chiocca EA, Aguilar-Cordova E, Hochberg FH (2004). The combination of adenoviral HSV TK gene therapy and radiation is effective in athymic mouse glioblastoma xenografts without increasing toxic side effects. Journal of Neuro-Oncology.

[B34] Urlinger S, Baron U, Thellmann M, Hasan MT, Bujard H, Hillen W (2000). Exploring the sequence space for tetracycline-dependent transcriptional activators: novel mutations yield expanded range and sensitivity. PNAS USA.

[B35] Qu Z, Thottassery JV, Van Ginkel S, Manuvakhova M, Westbrook L, Roland-Lazenby C, Hays S, Kern FG (2004). Homogeneity and long-term stability of tetracycline-regulated gene expression with low basal activity by using the rtTA2S-M2 transactivator and insulator-flanked reporter vectors. Gene.

[B36] Xu ZL, Mizuguchi H, Mayumi T, Hayakawa T (2003). Regulated gene expression from adenovirus vectors: a systematic comparison of various inducible systems. Gene.

[B37] Shockett PE, Zhou S, Hong X, Schatz DG (2004). Partial reconstitution of V(D)J rearrangement and lymphocyte development in RAG-deficient mice expressing inducible, tetracycline-regulated RAG transgenes. Molecular Immunology.

[B38] Freundlieb S, Schirra-Muller C, Bujard H (1999). A tetracycline controlled activation/repression system with increased potential for gene transfer into mammalian cells. The Journal of Gene Medicine.

